# V_H_H Structural Modelling Approaches: A Critical Review

**DOI:** 10.3390/ijms23073721

**Published:** 2022-03-28

**Authors:** Poonam Vishwakarma, Akhila Melarkode Vattekatte, Nicolas Shinada, Julien Diharce, Carla Martins, Frédéric Cadet, Fabrice Gardebien, Catherine Etchebest, Aravindan Arun Nadaradjane, Alexandre G. de Brevern

**Affiliations:** 1 INSERM UMR_S 1134, BIGR, DSIMB Team, Université de Paris and Université de la Réunion, F-75015 Paris, France; poonam.vishwakarma@univ-reunion.fr (P.V.); akhila.melarkode-vattekatte@univ-reunion.fr (A.M.V.); julien.diharce@univ-paris-diderot.fr (J.D.); cmartins@insa-toulouse.fr (C.M.); catherine.etchebest@univ-paris-diderot.fr (C.E.); aravindan.nadaradjane@univ-reunion.fr (A.A.N.); 2 INSERM UMR_S 1134, BIGR, DSIMB Team, Université de Paris and Université de la Réunion, F-97715 Saint Denis Messag, France; frederic.cadet.run@gmail.com (F.C.); fabrice.gardebien@univ-reunion.fr (F.G.); 3 3 SBX Corp., Tokyo-to, Shinagawa-ku, Tokyo 141-0022, Japan; shinada@sbx-corp.com; 4 PEACCEL, Artificial Intelligence Department, Square Albin Cachot, F-75013 Paris, France

**Keywords:** antibodies, frameworks, Complementarity Determining Regions, single-domain antibody, secondary structure, sequence–structure relationship, homology and comparative modelling, threading, deep learning, docking

## Abstract

V_H_H, i.e., VH domains of camelid single-chain antibodies, are very promising therapeutic agents due to their significant physicochemical advantages compared to classical mammalian antibodies. The number of experimentally solved V_H_H structures has significantly improved recently, which is of great help, because it offers the ability to directly work on 3D structures to humanise or improve them. Unfortunately, most V_H_Hs do not have 3D structures. Thus, it is essential to find alternative ways to get structural information. The methods of structure prediction from the primary amino acid sequence appear essential to bypass this limitation. This review presents the most extensive overview of structure prediction methods applied for the 3D modelling of a given V_H_H sequence (a total of 21). Besides the historical overview, it aims at showing how model software programs have been shaping the structural predictions of V_H_Hs. A brief explanation of each methodology is supplied, and pertinent examples of their usage are provided. Finally, we present a structure prediction case study of a recently solved V_H_H structure. According to some recent studies and the present analysis, AlphaFold 2 and NanoNet appear to be the best tools to predict a structural model of V_H_H from its sequence.

## 1. Introduction

**Figure 1:**
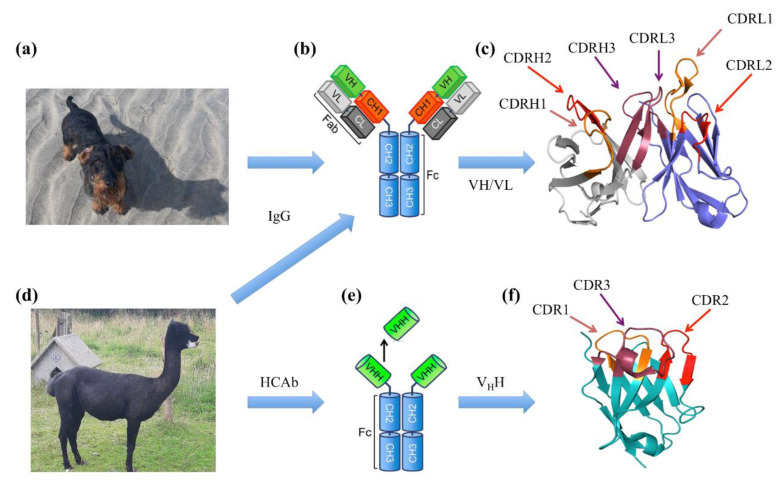
Antibody domains. Immunoglobulins are found in mammals such as the dachshund (*Canis familiaris*) named Snoopy (**a**) and a Vicuña (**d**); both pictures were taken in Normandy (France) in August 2021. The immunoglobulins are shown by domains in (**b**) Classical Immunoglobulin Gamma (IgG) and (**e**) HCAb (only found in the *Camelidae* family). (**c**,**f**) Antigen-binding domain of IgG and HCAb, namely the VH/VL domain and V_H_H domain. The FRs in the VH domain are shown in grey, in the VL domain in dark blue and in the V_H_H domain in cyan. The CDRs of the heavy chain are represented as CDRH1, CDRH2 and CDRH3, while CDRL1, CDRL2 and CDRL3 are CDRs of the light chain. The CDRs are represented in red shades: CDR1 (orange), CDR2 (red) and CDR3 (raspberry). Visualisation was performed using PyMOL [1,2,3]. Please notice that Snoopy was not harmed when taking these pictures and received an optimised dose of beef and turkey dog treats that he likes a lot.

Proteins carry out most of the functions in a cell. Among them, antibodies (Abs) or Immunoglobulins (Ig) play a major role in the immune response. The antibodies are found in mammals (see Figure 1a) and are classified into five main classes, namely IgA, IgG, IgD, IgE and IgM, IgG being the most abundant Immunoglobulin. IgM and IgA are multimeric Abs, while IgG, IgE and IgD are monomeric.

They all contain four chains: two identical heavy chains and two identical light chains. The heavy chains comprise three constant domains (CH_1_, CH_2_ and CH_3_), followed by one variable domain (VH). In contrast, each light chain has only one constant domain (CL_1_) and one variable domain (VL, see Figure 1b). The major function of antibodies is antigen binding, i.e., the capacity of recognising and binding to a specific target determined by the sequence and structural characteristics. The anatomy of the variable region (VH and VL) of an antibody can be further detailed with (i) four Framework Regions (FRs) and (ii) three (hyper)variable loops named Complementarity Determining Regions (CDRs) (see Figure 1c). CDRs constitute the main part of the so-called paratope region and are directly implicated in the interaction with the epitope (part of the antigen specifically recognised by the antibody). In terms of affinity, interaction ranges are in the nanomolar to micromolar range. This high affinity between a molecule and its antibody has led to a number of applications for diagnosis, therapeutics and in research fields [4,5,6]. One of the most interesting developments is the emergence of bispecific antibodies (BsAbs). BsAbs neutralise two specific targets using two different epitope binding regions formed by two variable fragments from each chain. Three of them are available on the market [7]: (i) Catumaxomab is used against chemotherapy-refractory ovarian cancer and recurrent malignant ascites by targeting EpCAM antigen on tumour cells and the CD3 antigen on T cells [8]; (ii) Blinatumomab against Philadelphia chromosome-negative (Ph-)-B-cell acute lymphoblastic leukaemia, which acts on CD3 and CD19, and a complete remission can be achieved (which was prevented before the approval of this drug) [9] and (iii) Emicizumab is used for the treatment of patients with congenital factor VIII deficiency [10].

Even though promising therapeutic strategies have been developed and are available in the market, Abs remain quite challenging to tackle in terms of manufacturing and purification [11,12]. One of the important limitations lies in the process cost to obtain a stable and functional molecule. Other factors like stability, bioavailability and the expected immune response are also difficult to optimise [13]. Hence, one relevant strategy to optimise the cost of production consists of using in silico approaches beforehand. Since the precise 3D structure plays a major role in the specificity of the recognition, a key step requires the elucidation of this 3D structure. Besides experimental procedures, theoretical methodologies constitute an alternative and promising way for predicting and studying their structures. Moreover, they are extremely valuable tools for understanding more deeply their properties and also offer the possibility to design new antibodies [14,15,16]. In this context, the analysis and prediction of IgG structures and complexes has been a major research field for 30 years. Two consecutive competitions, namely AMA-I and –II (Antibody Modelling Assessment), were initiated in the 2010s in order to assess the antibody structure prediction methodologies [17,18]. Their evaluations of state-of-the-art software like Accelrys [19], PIGS [20] and the Rosetta Antibody modelling suite [21] underlined the difficulty in properly predicting the conformations of the six CDRs (three from heavy chains and three from light chains) [17,18].

Interestingly, some animals have developed immunoglobulins with slightly different architectures that often encompass only one chain. The first one comes from cartilaginous fishes such as sharks. They have a special antibody called the “New Antigen Receptor antibody” (IgNAR). The IgNAR is longer than human IgG heavy chains, with dissimilar sequences [22]. The variable domain of the IgNAR is stable and small in size, which is very valuable for drug discovery [23]. Nearly thirty years ago, in addition to classical antibodies, a new class of immunoglobulin was discovered in camelids (this family includes Bactrian camel, dromedary camel, guanaco, llama, alpaca and vicuña; see Figure 1d) [24,25], where the light chain and, also, the CH_1_ domain of the heavy chain were absent. Consequently, they were named Heavy Chain-only Antibodies (HCAb, see Figure 1e). Interestingly, their VH domain, named V_H_H for Variable domain of the Heavy chain of HCAbs or commercially nanobody, is composed of, as any VH or VL domain, four frameworks and three CDRs (see Figure 1e). They are less than 130 residues long but often have a longer CDR3 than their conventional equivalents. Their smaller size makes them easier to manufacture (and at a low cost) than Abs. Moreover, V_H_H can be used independently from HCAb, while VH and VL must be combined for classical Abs [26,27].

V_H_Hs are thermostable, robust and can be tailored depending on the goal of their utility [28,29,30,31]. Furthermore, they bring advantages similar to classical Abs: they can be used for in vivo and in vitro diagnosis, therapy and research [31], e.g., in medicine [32], CAR-T [33], cancers [34,35,36,37,38,39,40], the detection of pathogens [41] or as biosensors [42]. For in vivo diagnosis, V_H_Hs have the capacity to pass through the blood vessels and diffuse rapidly to the tissues, which is helpful for in vivo imaging techniques like SPECT (single-photon emission computerised tomography) and PET/CT (positron emission tomography/computerised tomography). In terms of diagnosis, V_H_Hs have been efficient in diagnosing and monitoring the evolution [42] of HER2-positive breast carcinoma, atherosclerotic plaques and arthritis. V_H_Hs are also adapted for enzyme-linked immunosorbent assays (ELISA), a technique that is used for diagnosis and research purposes [43,44,45] for quantifying the amount of molecules in a biological sample. Another example is the Double-Antibody Sandwich ELISA (DAS-ELISA), where V_H_Hs are used to capture and detect the presence of a molecule of interest, e.g., to detect the alpha serum protein in foetal blood [46] or Staphylococcal enterotoxin C in dairy products [47].

V_H_Hs are also highly promising therapeutic molecules [48]. Yet, a humanisation step is first required to make V_H_Hs an effective therapeutic molecule for humans [49]. As an example, Caplacizumab was the first therapeutic V_H_H authorised by FDA against acquired thrombotic thrombocytopenic purpura (aTTP) [50]. Ozoralizumab and vobarilizumab are two other V_H_Hs currently utilised in clinical trials for rheumatoid arthritis [51,52]. Very recently, in the context of the COVID-19 pandemic, an impressive number of V_H_Hs have also been developed against the SARS-CoV-2 spike protein [53,54,55,56,57,58,59,60,61,62,63,64,65]. Therefore, a large number of pharmaceutical companies are now developing V_H_Hs. For instance, AbLynx (now part of Sanofi) has a maximum of six V_H_Hs in different phases, and three out of six are bispecific V_H_Hs [31].

The production of V_H_Hs is mainly obtained from immunising the response of camelids: the molecule of interest is injected to the animal, and the produced clones of V_H_Hs are then recovered. Specific sequences of V_H_Hs (e.g., by using next-generation sequencing) targeting a given antigen have to be determined, and the best proper clone(s) need to be expressed. In this aim, different methods have been developed [66] that use, for instance, phage display libraries or even plants [67,68,69]. For one particular antigen, a large number of experiments can be required to identify a lead V_H_H with a pertinent affinity.

As for classical Abs, the understanding of the interaction between V_H_H and its partner of interest is better achieved when the 3D structure of the complex is available [70]. Indeed, it would allow designing new V_H_H sequences with improved affinity or specificity or, in the context of biotechnology, grafting other partners (including V_H_Hs) onto it. Despite the increasing number of V_H_H structures available in the Protein Data Bank (PDB) [71] in recent years, the structures of a large number of V_H_H sequences are far from being fully solved. It is therefore necessary to use computational approaches to access this 3D information of interest [72]. However, as was shown by the first modelling of a V_H_H targeting the human DARC (Duffy Antigen/Receptor for Chemokine, now called Atypical Chemokine Receptor 1) protein in 2010, getting a relevant V_H_H 3D model [73] is quite difficult and remains extremely challenging, as exemplified by a very recent study [74].

While AMA-I and –II competitions have analysed IgG molecular modelling success, none have been done for V_H_Hs [17,18]. Similarly, the modelling of IgG structures benefits from a large number of dedicated tools such as PyIgClassify to classify the structures of their CDRs [75], but nothing equivalent exists specifically for V_H_Hs. For example, no dedicated approach to specifically model V_H_Hs had been developed until last year. Accordingly, for the first time, the present review aims at giving an overview of the specificities of the V_H_H 3D model proposals and their improvements over the last 12 years. Different tools available will be presented, and examples from the literature will be discussed. The advantages and performances of the modelling tools can be more easily compared and discussed, since they are applied to the same family of interest.

## 2. V_H_H Modelling

### 2.1. General Principle, a Short History

The evolution of sequencing techniques over the last 50 years implies that entire genomes are now accessible in a few hours, as is their proteome [76,77]. The gap between the number of protein sequences and experimental structures continues to grow despite the increased number of structures deposited in the PDB [71,78,79]. Additionally, for more than 40 years, methods for predicting 3D structural models from a sequence using computational approaches have made impressive progress [80,81]. The first approaches were based on simplified physicochemical principles, such as lattice folding in the early 1970s [82]. They were quickly supplanted by the use of analogies between proteins based on evolution. This paradigm states that mutations within a sequence could happen and accumulate themselves, leading to a minor modification in the 3D structure while keeping the same function. This conclusion gives rise to the following principle: “structure is more conserved than sequence”. These homology or comparative modelling approaches have been developed from the 1990s onwards [83] with the famous software called “Modeller” [84]. Nonetheless, the main pitfall of this approach lies in the threshold of the sequence identity between the query and the template sequences. When it is too low, it precludes the use of this kind of method. A solution can be found by looking for the compatibility between known folds and very distant sequences, namely the threading approach. These techniques had some nice results in the late 1990s, e.g., GenThreader [85], or, more recently, ORION [86,87], despite a higher computational cost. In parallel, approaches that combine small fragments from very distant proteins and optimise tens of thousands of 3D models are being developed and are reaching maturity with Rosetta [88,89] and I-Tasser [90]. Finally, very recently, Deep Learning approaches have allowed reaching a new level, sometimes leading to models of almost atomic quality with AlphaFold 2 [91] and trRosetta [92,93]. These approaches, composed of multiple complex neural networks, combine very long-distance evolutionary searches and advanced local compositional proposals. These advances have been achieved thanks to the rising computational power of GPUs in the last few years and better mathematical representations [94].

An important point to consider is the need for accurate metrics to quantify the distances between 3D structures (or structural models) to estimate the reliability of these predictions. The most classical, but also the strictest, is the Root Mean Square Deviation (RMSD) [95], as it corresponds to a classical Euclidean distance between two structures. Its evaluation therefore depends on the length of the protein of interest. To take into account the inherent flexibility of proteins (a long and poorly structured loop can lead to a high RMSD, despite a good overall prediction), many metrics weighting these atomic distances have been developed, the best known being the GDT_TS [96] and the TM-Score [97,98]. They take into account the quality of the folding compared to the reference structure and are thus very much used in the evaluation of Critical Assessment for Structure Prediction (CASP) competitions [99].

### 2.2. Abs and V_H_Hs Specificities

**Figure 2:**
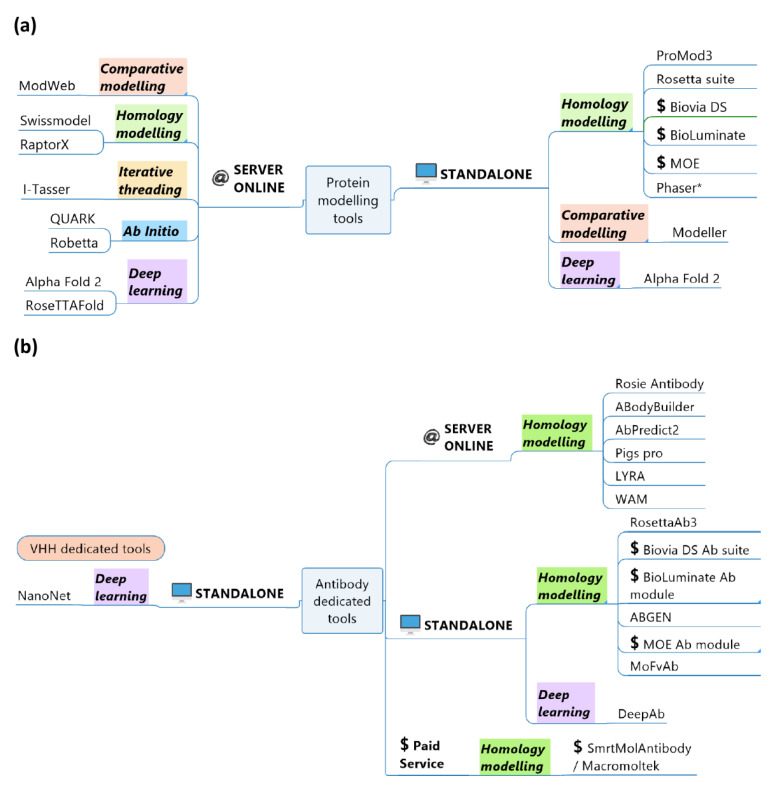
Protein structural prediction tools. The above representation is a summary of (**a**) general protein modelling tools and (**b**) Antibody and V_H_H modelling tools. All the tools are classified according to their availability (standalone and/or online server, academic or commercial) and the methodologies used (comparative/homology modelling, threading, ab initio and deep learning). * The phaser is dedicated to molecular replacement [105].

As presented before, Abs and V_H_Hs have specific global and local topologies, i.e., FRs and CDRs [100,101,102], or a VH/VL interface [103,104]. Hence, some specific Abs modelling tools have been developed. Of course, classical protein modelling tools (see Figure 2a) can be used for Abs and V_H_Hs. However, because of the various implications of Abs in biotechnology and biomedical domains and their diversity (Figure 2), the need for dedicated Abs tools has emerged (see Figure 2b). Abs software can model classical antibodies, but some of them offer options to model heavy chains or light chains only. V_H_H sequences are often compared to heavy chains of antibodies with lengthy CDR3 sequences. These tools can accept a V_H_H sequence and predict an associated structural model. In this review, modelling software programs are classified into simple and more advanced techniques. The number of V_H_H structural models remains limited in the literature, but their variety is quite rare and remarkable.

### 2.3. Modeller and ModWeb

Modeller [84] is probably the most important software of comparative/homology modelling. Developed in 1993, it is still maintained (https://salilab.org/modeller/, last accessed date: 7 February 2022), free for academics and included in commercial packages. Modeller automatically produces a model encompassing all nonhydrogen atoms based on (multiple) sequence alignment for a sequence (provided by the user) to be modelled with known related structures [106]. It takes as the input an alignment file of the sequence query with one or several sequences of structural template(s). Different refinement options exist. After multiple cycles of building and model evaluations, the chosen number of structural models is produced. The user must choose the best one associated with the lowest DOPE score [107]. Moreover, developers have proposed an online web server named ModWeb that uses PSI-BLAST [108] and IMPALA [109] to find compatible structural templates and Modeller to build the structural model [84].

The V_H_H fold looks well-conserved in the frameworks [70] but quite variable in CDRs. Comparative modelling using Modeller is a legitimate solution to predict the unknown structure of an antibody [110] and of V_H_H from its sequence. Nonetheless, the high sequence variability at the CDRs makes it a difficult challenge. Nowadays, Modeller is the most used software to model V_H_H structural models in theoretical studies.

In 2010, our team modelled the first V_H_H [111]. This V_H_H was optimised against human DARC/ACKR1, which is the entry point of a malarial parasite, *Plasmodium vivax*, into red blood cells. The structure of this anti-DARC V_H_H (named CA52) was built using two templates (PDB IDs: 1OP9 and 1JTO), selected using PSI-BLAST against PDB. The sequence query is 124 amino acids long and has an extra disulphide bond between CDR1 and CDR3 and was constrained as found in the second template. Two hundred models were generated using Modeller. Comparisons of the models were done by superimposition; the model with the median RMSD was selected. This un-classical approach was chosen, as the DOPE score was non-discriminative enough due to the extremely high structural similarity of the models. Indeed, this high similarity is explained by two reasons: the extra disulphide bridge constraint and an entirely equivalent fold of the frameworks [73]. This model was compared to other (and distinct) V_H_H clones obtained from the same camel and helped to search for a peptide able to bind DARC; an analysis of the electrostatic surface determinants was particularly important [112].

Another example focused on the cytokine TNF, a well-known drug target for several inflammatory diseases, such as Crohn’s disease. Two anti-human TNFR1 V_H_Hs were experimentally generated and linked with an anti-albumin V_H_H to create “TNF Receptor-One Silencer” (TROS) [113]. The two V_H_Hs were built with Modeller using multiple templates from four different structures (PDB IDs: 4FZE, 4JVP, 2KH2 and 3P0G). RAMPAGE software, a tool based on the local protein geometry, was used to validate the generated models [114]. The best models ranked were used for docking using ClusPro [115] with its human target TRNF1. This last step revealed the different binding sites of the two V_H_Hs. The authors used it to design bispecific V_H_Hs and tested them experimentally [113].

Other examples were done using the single structural templates for the design of V_H_H focusing on several targets: vascular endothelial growth factor receptor 2 (VEGFR2) for antiangiogenic strategies in cancer therapy [116], bone morphogenic factor 4 (BMP4, implicated in carcinogenesis and tumour progression [117]), CD47 molecule, a promising cancer biomarker [118] and Vascular Endothelial Growth Factor 165 implicated in tumour angiogenesis and metastasis [119]. One can also cite their use for an antivenom therapy inhibiting two myotoxic phospholipases from *Bothrops jararacussu* venom: Bothropstoxin I and II [120]. Furthermore, multiple templates were employed to model a V_H_H against matrix metalloproteinase 8 (MMP8) linked to several pathological conditions, e.g., lethal hepatitis and the systemic inflammatory response syndrome [121]. All these studies modelled a V_H_H structure from its sequence and used it to perform docking [122]. Studying the interaction and affinity between V_H_H and its target of interest was the major goal of these papers. Additional experimental information allowed refining the models and/or docking, e.g., V_H_H anti-MMP8 second poses were considered better in regards to ELISA data [121] and the development of a structure-based engineering approach to abrogate pre-existing antibody binding in Biotherapeutics [123]. Regarding the actual sanitary context, Modeller was used for the design of a V_H_H that neutralised SARS-CoV-2. The docking made with the conceived V_H_H was in excellent agreement with the experiments and explained the difference of binding between V_H_Hs [61].

Modeller was also used for the refinement of small-angle X-ray scattering analysis results in the context of the interaction of a V_H_H interacting with the Disrupted-in-Schizophrenia 1 (DISC1) protein, involved in neurodevelopment and chronic mental illness. Modeller, a more supervised approach, was shown to be more efficient than a more complex method, QUARK (detailed later) [124].

### 2.4. ABGEN

ABGEN software was made available in 1996. ABGEN is a homology-based algorithm that models an antibody and has some strong similarities with Modeller. It consists of two modules called ABalign and ABbuild [125]. ABalign finds templates for the heavy and light chains and provides an identity score for each template identified. Based on the best template selected, ABbuild constructs a rigid model for all chains; then, the loops and side chains are optimised. Finally, ABGEN includes dedicated mechanistic approaches with XPLOR [126] and GROMOS [127] to refine the obtained models. ABGEN is no longer accessible and has not been used on V_H_Hs but was the first to specifically optimise the CDR loops.

### 2.5. Web Antibody Modelling

Web antibody modelling (WAM) was a very interesting hierarchical approach published in 2000 [128] and dedicated to antibodies. It improved previous works [129,130] and can be described in five consecutive steps:

1. A similarity search is performed to build the framework (backbone and side chains) and canonical loop backbones with closer structures (in terms of sequence similarity).
2. Using CONGEN [131], the canonical loop side chains are constructed using an iterative placement technique searching for the global minimum energy conformation.
3. Depending on the loop length, alternative conformations are produced by using directly the PDB or CONGEN again.
4. A specific solvent-modified version of the Valence force field is tested to assess the different conformations.
5. Finally, the conformation is selected from the five lowest energy conformations. The final model is the conformation with the set of torsion angles closest to the canonical one, as defined in the chosen clustering approach.

Theoretically, this tool can be used to model a V_H_H sequence, but this has not been done, and its website has been unavailable for some years. Still, it represents the first real attempt to optimise part-by-part an antibody.

### 2.6. SWISS-MODEL

SWISS-MODEL is a famous online protein homology modelling server that was firstly published in 2003 [132]. It has been frequently updated with both interface ergonomic and methodological improvements [133,134], but the basis is rooted in the principles of the Modeller algorithm (see above). The research of compatible sequences is made by PSI-BLAST [108], combined with delicate HHblits [135]. Then, the user can select which template(s) to use. An interesting point is the different measures provided to assess the qualities of the several models built, i.e., the Global Model Quality Estimate (GMQE) [133]. Building of the structural model is done with locally developed OpenStructure, i.e., an integrated software framework for computational structural biology [136]. In a similar way, they also developed measures to assess the quality of the models, i.e., QMEAN scoring function [137]. While it is a generic approach, it has been used for the modelling of different classical antibodies [138,139,140,141].

Their in-house software ProMod3 was recently provided freely to download as a standalone package [142]. It can be used to identify structural templates after sequence alignments for a given V_H_H sequence. Then, it produces multiple homology models in batch mode.

SWISS-MODEL was used to generate structural models of humanised V_H_Hs. Murakami and co-workers used SWISS-MODEL directly to generate their humanised V_H_Hs and later relaxed the 3D structural models with advanced molecular dynamics (MD) simulations. They previously classified CDR3 conformations into four clusters carried out on MD to see their stability. Indeed, their clusters seemed highly biologically relevant [143]. SWISS-MODEL was also used to generate a structural model of a V_H_H that binds to the famous spike protein of SARS-COV-2. This particular V_H_H was used to understand the structural basis for the induction of a spike trimer [144]. Noorden and co-workers built a complex of a V_H_H and SAN2 protein and provided further comprehension of a nuclear pore complex structure [145]. Thanongsaksrikul and co-workers developed libraries of VHs and V_H_Hs against botulism. They succeeded in the identification of Abs against the light chain of type A *Clostridium botulinum* (BoTxA/LC) neurotoxin. Using SWISS-MODEL, they obtained VH and V_H_H structural models. The interface binding between BoTxA/LC and the selected VH/V_H_H was determined by using ZDOCK and RDOCK modules on Discovery Studio 2.1 (Accelrys Inc., now the Biovia-Dassault System). As they noted, “BoTxA/LC neutralization by the V_H_H should be due to the V_H_H insertion into the enzymatic cleft of the toxin, which is usually inaccessible to a conventional antibody molecule” [146]. Higashida and Matsunaga modelled all their V_H_Hs with SWISS-MODEL before doing advanced molecular dynamics, especially to look at the important parameters to properly gain the inner flexibility of V_H_H CDR3 [147]. Orlov and co-workers also modelled a V_H_H with SWISS-MODEL to gain the structural basis of V_H_H recognition of grapevine fanleaf virus implicated in the virus resistance loss. The proposition of this structural model allows a better understanding of the epitope and helps to design experiments to confirm it [148]. Mahajan and co-workers used SWISS-MODEL V_H_H structural models (refined by molecular dynamics) in structure-based computational methods to optimise the binding affinity of the non-amyloid component of human α-synuclein, a natively disordered protein implicated in the pathogenesis of Parkinson’s disease (with a large number of experiments) [149].

### 2.7. MoFvAb

MoFvAb was published in 2015 [150] and focuses on “Modelling the Fv region of Antibodies”. In the first step, it annotates FRs and CDRs using the WolfGuy numbering system [151]. Based on the segments, templates are identified for heavy and light chains. For all chains, rigid models are produced, except for CDR-H3. The latter undergoes de novo building. In the next step, the CDRs are relaxed, and a side chain refinement is performed based on the neighbourhood algorithm. The final steps involve VH/VL orientation prediction and refinement, rotamer optimisation and CHARMM minimisation [150]. The methodology was tested on the AMA-I and -II datasets with good results but was never applied on V_H_H. It might be adapted for V_H_H, but the approach is not currently available.

### 2.8. Prediction of Immunoglobulin Structure

These web servers were developed by Anna Tramontano’s group. Anna Tramontano worked previously on antibody structures, sequence–structure relationship and modelling using classical approaches [152,153,154,155]. The first version named “Prediction of ImmunoGlobulin Structure” (PIGS) was published in 2008 [20], as the authors found that WAM had numerous limitations and was not very flexible. On the basis of the results from previous research, CDRH3 was modelled differently according to its length [156]. PIGS proposed structural models of the complex VH/VL for the user-provided sequences. The evaluation of the approaches was tested in the original paper but also in AMA-I and -II competitions, with good results [17,18]. The second version, named PIGS PRO, was proposed in 2017 [157]. As WAM, it can be divided into five steps:

1. Frameworks are prepared: Structural templates for the frameworks are selected using sequence identity with protein structures from PDB.
2. Five of the six CDRs are built: CDRs L1–L3 and H1 and H2 are modelled by getting conformations from antibodies with the same canonical structures discarding the identity sequence.
3. To complete, a CDRH3 loop is proposed using the structural template with the highest sequence identity with the query sequence.
4. All is merged: The complex VH–VL is modelled.
5. At last, SCWRL is used to optimise the side chain conformations of the predicted model.

Interestingly, the user had the possibility of providing a V_H_H sequence instead of a VH sequence by putting in a “fake” canonical VL sequence to initiate the prediction job. This V_H_H/VL structural model could have provided an interesting approach. The web server is not currently online.

### 2.9. AbodyBuilder

ABodyBuilder is a dedicated tool developed in Deane’s lab for antibody structure prediction. The algorithm first annotates and finds templates for VH and VL alone and for the complex VH–VL [158]. A new update has been deployed, and it is now also usable for V_H_H alone [159]. The approach is hierarchical, as often seen. The FREAD algorithm tries to identify the templates for CDRs. If no templates are found, then the Sphinx loop is triggered ab initio. The final step is to use PEARS to predict the side chain conformations using the IMGT position-dependent rotamer library. An interesting feature is the estimation of the expected RMSD with a confidence value of the resulting antibody model [158]. As a valuable example, this method has been used recently for the analysis of SARS-CoV-1-specific V_H_H to apprehend the conformational diversity of the CDR region, its affinity and stability [160].

### 2.10. LYmphocyte Receptor Automated Modelling

LYmphocyte Receptor Automated modelling (LYRA) was an improved approach to predict B- and T-cell receptor structural models [161], presented as an online web server (https://services.healthtech.dtu.dk/service.php?LYRA-1.0, last accessed date: 8 February 2022). The authors have done important developments regarding curation both in terms of sequences and structures. The sequences have been annotated and numbered with the Kabat–Chothia approach adopted by Abhinandan and Martin [162] and reduced in terms of redundancy. Research of the compatible optimal sequences was done using a Hidden Markov Model [163]. The best-scoring profile is used to deduce the receptor (TCR or BCR) and chain type (heavy or light). At this stage, the alignments are recertified to correctly identify the heavy and light chains. An important point is the generation of different variations of the scenario by looking at (most of) 20 different structural templates to ensure there are no clashes. Similarly, to refine it, CDRs are searched on a defined library based on a specific CDR clustering [164]. Finally, to create the final model, the templates from both chains are assembled, and the side chains are repacked [161]. This job process is fast and takes less than a minute on average. The user can replace the heavy sequence with the V_H_H sequence and get a model for this V_H_H sequence in a similar way to PIGS and PIGS PRO.

### 2.11. Phyre2

Phyre [165] and its successor Phyre2 [166] are generic approaches very similar to techniques such as SWISS-MODEL that combine evolutionary information with a dedicated analysis of the protein structure dataset. It is an available online web server (http://www.sbg.bio.ic.ac.uk/~phyre2/html/page.cgi?id=index, last accessed date: 9 February 2022). Phyre2 has two modes: a normal and an intensive mode.

The normal mode consists of three steps:

1. Related sequences (with or without structures) are detected by HHblits [135] and HHsearch [167] to produce multiple-sequence alignment and to search for adequate templates for the input sequence. Once the templates are identified, the models are constructed but roughly only with the backbone.
2. A specific step is done to optimise the loop modelling where the indels (insertions and deletions) in these models are found.
3. The side chains are grafted to build the final Phyre2 model.

The intensive mode has two steps. The first step produces many alternative models of the same query with the basic mode. Different metrics are then tested related to the confidence interval and coverage. When a region is considered not covered by “homologs”, then the Poing algorithm enters the stage to predict the structure using ab initio components. This algorithm mimics a virtual ribosome by performing a folding process only with Cα atoms. It focuses at first on repetitive structures, then the loops [168]. At the end, the backbone is completed with the Pulchra algorithm [169], and the side chains are finally added with R3 [170], such as for the normal mode.

Different V_H_H structural models were generated with Phyre2. A first example implies Urease C (UreC) of *Helicobacter pylori* has an important role to play in bacterial colonisation of the gastric mucosa. Loss of its activity has been speculated to arrest *H. pylori* colonisation, i.e., a pertinent target for therapeutic intervention. Different clones of V_H_H were produced against UreC. A high-affinity V_H_H, called HMR23, was optimised and selected for further analysis. To understand the difference of binding properties between parent anti-UreC V_H_H and HMR23, structural models were generated using Phyre2. The modelling of parent and mutant V_H_Hs was essential to understand the structural conservation in terms of fold in the latter [171].

A second example focuses on *Acinetobacter baumannii*, a multidrug-resistant bacterial species responsible for many hospital-derived infections. Its ability to form biofilms helps its survival in hospital conditions. Inhibiting the formation of biofilms can contribute to the reduction of infection. In a previous study, researchers detected an efficient V_H_H clone against the Biofilm-associated protein (Bap) of this bacterium [172]; the purpose of this study was to go further using 3D structural information. An interesting point to notice is the use of multiple methodologies to propose the structural models. They were produced with (i) Phyre2 [166], (ii) the Protein Structure Prediction Server (PS^2^V^2^) [173] that builds the models with Modeller [84] and (iii) LOMETS [174]; the last two approaches are methodologically equivalent. A total of ten models for each template were generated with ten distinct structural templates. Model evaluations were done using ProSA [175,176] and then further refined using Modrefiner [177] to select the best ones. It was one of the few studies where multiple approaches were really tested. Then, they performed a docking analysis with ZDOCK [178,179]. Hence, the authors defined important V_H_H residues and their interactions for the ligand-binding site. They proposed binding modes between Bap and the V_H_H. Finally, the functional residues in the largest cleft implied in ligand binding were identified [180]. An identical approach was used for a V_H_H against chronic inflammatory disease caused by *Porphyromonas gingivalis* [181].

### 2.12. RaptorX

RaptorX is an automated server that performs template-based tertiary structure modelling [182,183]. It derives from RAPTOR, a classical threading approach [184,185]. The improvement of RaptorX versus RAPTOR consists in the use of a nonlinear alignment scoring function with a conditional random field (CRF), providing local entropy. In the first step, the sequence is cut into domains, and the predictions of different features, such as disorder prediction, are performed. Then, the templates are searched and ranked through a threading process. The alignment quality is assessed by Artificial Neural Networks. At the end, RaptorX provides multiple final models, including models that are produced based on multiple top-ranked templates. The latest version of RaptorX has now evolved and includes a deep convolutional neural field (DeepCNF) to predict the secondary structure [186]. DeepCNF has two components: a deep convolutional neural network (DCNN) and a conditional random field (CRF) for the input and label (output) layers that are CRFs. It has recently been enriched by the use of deep learning to predict protein contacts [187]. RaptorX [182,183,188] was used in exciting research on *Bordetella pertussis*. This aerobic, non-spore-forming, Gram-negative coccobacillus was implicated in the renewed outbreak of whooping cough in the elderly. Its adenylate cyclase-haemolysin toxin (CyaA) plays an important role during the early phase of infection. A specific subdomain named CyaA-RTX is involved in toxins binding to target cells. Through the screening of a VH/V_H_H phage display library, two VHs and two V_H_Hs clones were identified after several optimisation rounds [146,189,190,191]. Three-dimensional structural models of these proteins were built using RaptorX [192]. The loop conformations were obtained using the dedicated FALC loop modelling web server [193]. The best models were further validated using various algorithms available on the NIH SAVE server. Then, the best resulting models were well-refined and optimised by energy minimisation performed with Gromacs software [194]. Afterwards, in order to predict their mode of interaction, the V_H_H models were docked on the target CyaA-RTX domains with the ClusPro web server [115]. Interestingly, all the nanobodies were found to interact with the target, especially in the linker region between the two domains CyaA and RTX, i.e., in excellent accordance with the experimental data.

### 2.13. Rosetta, Robetta, Rosetta Antibody and V_H_H Modelling Application

Developed under the guidance of David Baker, the Rosetta story began in the last century. Originally, it mainly consisted in an ab initio approach [195], but progressively, it evolved towards a de novo strategy that combines small structural fragments obtained from a sequence search in a dataset. The addition of evolution information and local protein constraints improved the quality of the results [89,196,197]. A last round of various improvements led to erasing non-native contacts. Finally, an intensive production of alternative structural models and the clustering of models coming from homologous sequences contribute to make Rosetta a great successful tool in the proposition of structural models [198,199]. Rosetta development was impressive and can be analysed through different prims. RosettaCM, dedicated to optimise comparative modelling steps, competed with the best similar approaches [199]. A web server named Robetta is freely available (https://robetta.bakerlab.org/, last accessed date: 10 February 2022) [200,201].

This powerful tool is also freely downloadable for academics and can be installed locally. Rosetta software has evolved a lot since its beginning, being highly scriptable and customisable [93]. Besides 3D predictions, it encompasses extremely valuable services, e.g., protein design [202] or protein docking [203,204], which are of particular interest in the context of V_H_H exploration. Rosetta was applied for the study of an anti-glycoprotein (cAbAn33), anti-lysozyme (cAbLys3) and anti-prostate-specific antigen (cAbPSA-N7). The contribution to the stability of these proteins of an extra-disulphide bond (between CDR1 and CDR3) present in camels and llamas was more precisely explored. Fortunately, cAbAn33 and cAbLys3 have available protein structures that were used for creating models of mutants in which the extra cysteines CDR1 and CDR3 were replaced. ESyPred3D [200], i.e., a methodology based on Modeller and Robetta [200], was used to generate the mutant models. The authors mentioned that the models from ESyPred correspond to those generated by Robetta (data were not shown). Hence, molecular modelling in this study has been useful in understanding the structural conservation of mutants in terms of fold. The models of the mutants predicted had very few differences compared to their parent V_H_H structures [205]. From the generalist Rosetta software, a specific antibody development was done, namely RosettaAntibody [206]. This tool is available online and can be used through Rosie (https://rosie.rosettacommons.org/antibody/, last accessed date: 10 February 2022). The protocol consists of three steps for a given antibody sequence [21,207,208]:

1. In the initial step, the CDRs are identified using the Kabat CDR definition, and the residues are renumbered using the Chothia scheme. The template selection is then carried out for all the frameworks, and five of the six CDRs (CDRH1 and CDRH2 and CDRL1–CDR3),
2. From the selected templates, a preliminary model is created using homology modelling, as it was shown to be more accurate than a completely de novo approach.
3. CDRH3 de novo loop modelling completes the model prediction, along with the optimisation of the VH–VL interface.

As the Rosetta modelling suite also incorporates a docking approach, this last was optimised for antibodies [209]. However, RosettaAntibody appeared less efficient with single-chain antibodies and, so, with V_H_Hs. Accordingly, a specific adaptation in the loop definition was done [210] to better take account of the specificity of the V_H_H CDRs, especially CDR3 [211]. Until 2021, it was the only specific development and assessment dedicated to V_H_Hs. This study underlines the difficulty in V_H_H modelling and, specifically, the fact that an approach for IgG is not optimal for V_H_H. For a while, the specific script for V_H_H was not available in the new versions of RosettaAntibody, but it is now usable again. Even if the tool is not used directly, it has been frequently cited as an analysis of CDRs that is quite precise, i.e., for optimising synthetic V_H_Hs [212].

### 2.14. AbPredict2

AbPredict is implemented in the Rosetta modelling suite through two versions: ABPredict 1 [213] and ABPredict 2 [214]. It can be downloaded (http://abpredict.weizmann.ac.il/bin/steps, last accessed date: 14 February 2022) and is also free for academia. In short, AbPredict exploits known antibody 3D structures and optimally combines backbone conformations with a low-energy Monte Carlo search. A few details are given in the next section.

AbPredict methodology is based on five independent databases comprising backbone torsion angles of the segments of VH, VL, CDRH3, CDRL3 and rigid body orientation of heavy and light chains. The initialisation starts by combining random segments from the five databases. A biased simulated annealing Monte Carlo (SA-MC) sampling is performed, with thousands of independent trajectories. The lowest energy conformation is extracted from each of these trajectories. From the latter list of conformations, the final predicted antibody model is the one that has the lowest energy.

AbPredict has been benchmarked using the AMA-II antibody set and compared to the methods presented therein. It performed in the upper third of all the compared methods [208]. It was recently compared to RosettaAntibody and RosettaCM, a generic version, in the case of long CDRH3. Surprisingly for these very hard cases, RosettaCM was slightly better than the two other dedicated methodologies [215]. Thus, despite some success in Ab modelling, it seems that ABPredict is not used for V_H_H modelling nowadays.

### 2.15. Biovia Discovery Studio/Antibody Modelling

Discovery studio (DS) is a commercial toolbox coming historically from the Accelrys company located at San Diego, CA, USA (a merging of Molecular Simulations Inc., Synopsys Scientific Systems, Oxford Molecular, the Genetics Computer Group (GCG), Synomics Ltd. located at Witney, Oxfordshire, United Kingdom and some others) and is now owned by Dassault Systèmes Company. It was renamed BioVia. BioVia Discovery Studio uses Modeller at the backend to model protein structures from a user-provided sequence [84]. DS proposes different strategies based on homology modelling but with dedicated tools to model the antibodies named Biotherapeutics and Antibody modelling. They were positively tested in AMI-I and -II and validated [216].

The first and simplest strategy (named single template) consists of finding the best templates (based on the sequence identity) for the heavy and the light chains of a given antibody sequence. Both of these templates should belong to the same antibody structure. Spatial orientation or the interface between the heavy and the light chains will also be used as a template from the same antibody.

In contrast, in the second strategy (named as chimeric), the best templates for the heavy and light chains and the interface between the chains can come from different antibody structures. After identifying the top five best templates, five models are built using Modeller [84]. If the sequence identity between the template and the target is too low (inferior to 10%), the template is discarded. This initial step is followed by the CDR refinement of the top five models. The CDR regions are identified using the IMGT, Chothia, Honegger or Kabat numbering schemes [101]. The CDR templates are searched and ranked by sequence similarity according to the BLOSUM62 matrix and, finally, rated based on the crystallographic resolution. The CDR loops are built with Modeller using the best-ranked templates while maintaining the frameworks in these models.

Two V_H_H modelling applications have been done using DS. Jullien and colleagues worked on histones, proteins implicated in the nucleosome complex. Some posttranslational modifications of histones are indicators of gene expression. This feature can be exploited by binding exogenous proteins to these histones, which helps to understand the chromatin dynamics. In this study, V_H_H rose against the H2A–H2B complex in the chromatin, i.e., named chromatibody, and were experimentally tested and automatically modelled using Modeller from Discovery Studio. The best models were selected based on the Modeller scoring function (molpdf) and DOPE scores [107] and then refined. This molecular modelling has been helpful in suggesting the appropriate placement of the chromatin-binding motif in the β-hairpin region of the CDR3 into the H2A–H2B protein cavity (Jullien et al. 2016).

The second case dealt with a V_H_H that targets Phospholipase A2 (PLA2), an enzyme present in snake venom that digests cell membrane lipids. The number of snakebite cases, especially due to monocled cobras (*Naja kaouthia*) in Thailand, is on the rise. Hence, an antibody against PLA2 has practical interest, especially a camelid V_H_H, due to its thermostable properties. A humanised dromedary V_H_H phage library was previously obtained [146]. Two V_H_H clones from PLA2 bio-panning (P3-1 and P3-3) were selected for further studies of modelling and docking. Both V_H_H clones are classical V_H_Hs, along with P3-3, having an extra CDR1–CDR3 disulphide bond. The templates were selected using BLAST against PDB, and the structural models were built with DS. The steric hindrance of the models was assessed and, also, geometrical properties with the Ramachandran plot. The best models were selected for docking with PLA2 using ZDOCK. The docking experiments revealed that the V_H_H models bind to the Ca^2+^-binding site, the active site and the phospholipid-binding site of PLA2 [191]. Despite their interest, these findings have not yet been experimentally validated.

### 2.16. MOE/Antibody Pipeline

MOE belongs to the Chemical Computing Groups Company, located at Montreal, QC, Canada, and has a pipeline for antibody and biologics designs [217]. The tool works for classical antibodies and V_H_H. For a V_H_H sequence, MOE searches for templates in two databases: in-house-built and PDB databases. In the in-house-built database searching process, the template will be kept if the sequence similarity with the target is more than 50%. From the in-house-built and PDB databases, the top 10 and top five hits will be retained, respectively. The templates are then constructed by grafting the frameworks and the CDRs. Multiple models are built, and then, an energy minimisation is performed. From this group of models, a consensus model is established, and different optimised final models are proposed.

With the final model, CDR3 conformations are further explored and, lastly, clustered. A unique CDR3 conformation is finally grafted in the V_H_H model. Once again, an energetical evaluation is done. The top three models with the lowest binding energies will be the final output of this program [217].

Different studies have been made with classical antibodies. A recent study combined IgG and V_H_H as bispecific antibodies. MOE structural models were tested by molecular dynamics and analysed in light of the experimental data. They could compare different V_H_H behaviours and especially observed problematic interactions that were too strong between one IgG chain and one V_H_H [218].

### 2.17. Schrödinger BioLuminate and Antibody Pipeline

BioLuminate is a suite developed by Schrödinger Company, located at New-York, NY, USA. It is a pipeline for general homology modelling but includes a specific design for antibodies. The modelling protocol is:

1. Frameworks are detected in a curated antibody database, providing structural templates (selected using a sequence identity).
2. Next, a set of CDRs are grafted after scanning another custom database containing only CDRs and a selection based on structural clustering, sequence similarity and stem residue geometry matching.
3. In the final step, the in-house Prime software repacks the side chains and minimises the antibody model.
4. A CDR3 loop is built using the ab initio method.

Schrödinger does provide detailed information about BioLuminate’s Antibody modelling tool and tutorials: (https://www.schrodinger.com/training/building-homology-models-multiple-sequence-viewereditor214, https://www.schrodinger.com/training/antibody-visualization-and-modeling-bioluminate-workshop-tutorial214, last accessed date: 15 February 2022).

Their antibody pipeline was used in a study on *Clostridium difficile* Toxin (CDT). This last is a potent toxin responsible for antibiotic-associated diarrhoea. These toxins belong to the C2 class of toxins, which means the presence of two subunits (CDTa and CDTb). The CDTa subunit is responsible for the ADP-ribosylation of actin, and CDTb is responsible for forming a toxin–pore complex through which the CDTa subunit is internalised. This study attempted to inhibit the function of the CDTa subunit using V_H_H from lama. Three anti-CDT V_H_H clones were modelled using the BioLuminate package. Two of the clones had an extra disulphide bond that was preserved in the structural models. These models were used in a docking experiment to understand their precise mode of interaction with their target. The models were docked onto CDTa using the PIPER module in Discovery Studio. All three V_H_Hs were found to bind to the NAD^+^-binding cavity in CDTa. Two compete with each other, whereas the last has a different binding site. This study also reported interactions in FRs of the V_H_H used to lead to a better understanding of the experimental data.

### 2.18. Macromoltek’s SmrtMolAntibody

The Macromoltek Company, located in Austin, TX, USA, created SmrtMolAntibody. The first step of this algorithm is to search an antibody database to detect templates for the VH and VL query sequences with the BLAST approach [219]. Then, a similar search is done on a loop database to find more adequate templates for all six CDRs. Based on the identified templates, assembling the frameworks and grafting the CDRs allow building an initial antibody model. A specific modelling step is done for the CDR(H)3 loop. The top 50 models with the best (energy) CDRH3 loop conformations are retained. For each of these 50 models, the side chains are repacked, and their torsions are minimised. The above-mentioned final steps are subject to multiple evaluations based on an energy score with modified and softened Lennard-Jones associated with side-chain rotamer frequencies [219].

### 2.19. I-TASSER and C-I-TASSER

During multiple rounds of CASP, with David Baker’s Rosetta, I-TASSER [90] was the best available method to propose pertinent structural models [220]. I-TASSER is based on Local Meta-Threading Server (LOMETS) [221]. LOMETS exploits deep learning-based threading methods and profile-based programs to identify the best templates and to annotate structure-based protein functions. Once the templates are identified, I-TASSER uses a Monte Carlo-based simulation to produce and to refine the protein structural model. The latest development includes deep learning-based contact predictions in the I-TASSER methodology, which is now named C-I-TASSER [222].

The I-TASSER web server was used to model different V_H_Hs. A first study looked at the hepatitis C virus (HCV). This virus has six non-structural proteins (NS). Fusion proteins NS3 and NS4 form a serine protease, which cleaves the linker between NS5A and NS5B. This last is directly implicated in the virus life cycle and is a good target. Three V_H_H clones were generated against the fusion HCV protease NS3/4A. The I-TASSER web server was used to build the three structural models. They were refined using ModRefiner and fragment-guided molecular dynamics [177]. Computational docking analyses revealed that CDR2 and CDR3 of the three V_H_H would bind to the NS3/4A catalytic triad residues. Two V_H_Hs residues from FRs were also implicated in interactions with NS3/4A [189]. This analysis brought up a plausible explanation for better understanding the experimental data previously obtained.

The second example looks at Staphylococcal Protein A (PrA). It binds to the FC and sometimes Fab regions of antibodies and makes it an attractive tool for the in vitro isolation of antibodies. This study attempts to understand the binding of PrA to V_H_Hs. Fridy and collaborators designed V_H_Hs with minimal CDRs to remove their contribution in binding to PrA. The LaP-1 (llama antibody against Protein A) V_H_H was optimised, and a few mutants were selected [223,224]. The I-TASSER web server was used to model the structures of these V_H_Hs. The best models were chosen based on the c-score of I-TASSER. The models provided insights on the PrA binding to antibodies and, also, into the structural stability of the mutants and the parent V_H_H [225].

### 2.20. QUARK and C-QUARK

QUARK is a reference ab initio structural prediction strategy [226]. It is based on a force field composed of 11 energetic terms, classically representing three resolution levels (atomic, residue and topology). The strategy can be described in three stages.

Based on the input sequence, the secondary structures, torsion angles and solvent accessibility and generated fragments are predicted. Then, Multiple Replica Exchange Monte Carlo (REMC) simulations, using a semi-reduced protein model, are performed to build multiple alternative conformations. The final step uses SPICKER for clustering [227] and performs refinement on the full-atom structure.

The latest version of this software is named C-QUARK [228,229]. When compared to QUARK, two steps are added at the start: (1) DeepMSA-generated multiple sequence alignment [230] and (2) contact map prediction based on deep learning methods. Even if QUARK and C-QUARK performed well in CASP competitions [231,232], they were not used for antibody and V_H_H modelling.

### 2.21. AlphaFold 2

AlphaFold was developed by the industrial laboratory DeepMind [233]. It joined for the first time the CASP competition in 2018 (CASP13) [234] and won the Free Modelling category [235], i.e., to predict novel protein folds. The Template-Based Modelling category, i.e., protein folds already in the PDB, was won by Zhang’s group [90]. Two years later, they created a shockwave by greatly improving the quality of their methodology [91] and clearly won this CASP competition. Its inclusion of advanced deep learning approaches coupled with the GPU power of DeepMind allowed a fantastic success [236,237]. It reduced the percentage of the dark human proteome [238] and opened opportunities for drug design [239].

AlphaFold 2 uses multiple sequence alignment (MSA), residue pairing information and structural templates for a given sequence that should be modelled [91]. A transformer, called Evoformer, processes all of these inputs. An important innovation point about this latter is that it allows information exchange between the MSA and residue pairing blocks. The authors emphasised that this Evoformer block helps in establishing a rational between the spatial and the evolutionary information of residues. After passing through Evoformer, the resultant information is processed by structural blocks that output the coordinates directly. One particularity is that the intermediate MSA, residue pairing information and the predicted structure are reinjected into the Evoformer blocks. By default, these intermediate results are reintroduced in the network three times, which improves the predictions. AlphaFold 2 can be downloaded from a GitHub repository (https://github.com/deepmind/alphafold, last accessed date: 9 February 2022). However, users need good computers with performing GPU cards and a good amount of memory. Interestingly, an online Jupyter notebook can also be used (https://colab.research.google.com/github/sokrypton/ColabFold/blob/main/batch/AlphaFold2_batch.ipynb, last accessed date: 9 February 2022). It produces five models by default [240]. For each residue in the model, a confidence score is provided, and it is used to rank the models among them.

AlphaFold 2 was a hot topic for 2020 and 2021, leading to a revolution in protein structural model building [241]. However, AlphaFold 2 does not always give 100% correct/meaningful predictions, i.e., some globular proteins are still not properly modelled [242], and transmembrane proteins are not close to a near-native structure, as they are hard targets. Antibodies were not specifically tested, but different examples showed, as seen for Rosetta [207], that classical comparative modelling still performs better. It is so logical that no V_H_H modelling has been performed at this time.

### 2.22. RoseTTAFold and DeepAb

Deep learning has made a paradigm change in structural bioinformatics [243,244,245,246,247,248]. Therefore, the famous Rosetta has been upgraded and now leverages these new advances [201]. trRosetta is based on deep learning approaches with an all-atom energy function [249] combined with inter-residue distance constraints and orientation distributions [92,250].

Based on the properties and successes of AlphaFold 2 [91], Baker’s team extensively studied the different neural network architectures and proposed a new deep learning-based methodology called RoseTTAfold [251]. It is based on a three-track (1D, 2D and 3D) neural network, which can simultaneously process multiple sequence alignments (MSA), inter-residue contacts and refinement of the predicted structure. The connections in this network allow efficient learning about the relationships between the protein sequences, distances and the coordinates. The interlinked 1D and 2D neural networks take and process cropped MSA and templates as the input. The 3D track first provides a backbone-only model, and then, SE(3)-transformer performs an iterative refinement by using the inference of a relationship between the sequences and the templates. By the end of this stage, the user will have a full-atom model. The Robetta server with RoseTTAfold methodology provides predicted models with a confidence index (Å error estimate per position), which is used to rank the five final models [251]. As for AlphaFold 2, since the approach is extremely recent, no use in predicting the structure of V_H_Hs has been observed.

While preparing the submission of this review, DeepAb was published [252]. It can be considered as the natural evolution of RosettaAntibody with deep learning RoseTTAfold. The algorithm can be divided into two main steps. The first part has a deep residual convolutional network, which consists of 1D ResNet, a Bi-Long short-term memory (Bi-LSTM) repertoire encoder and 2D ResNet. The 1D ResNet and Bi-LSTM encoder processes structural and evolutionary features. The 2D ResNet will output inter-residue distances and orientations. In the second step, Rosetta minimisation is used with distance and angle constraints. Concerning V_H_H modelling, Ruffolo et al. observed that CDR3 resembles that of CDRH3 [252]. Nevertheless, they suggest that the methodology should be refined with a database dedicated to V_H_Hs. In the present stage, DeepAb seems to be more specific for classical antibodies than V_H_Hs.

### 2.23. NanoNet

NanoNet was proposed to the scientific community in August 2021 [253]. It is truly the first optimised V_H_H approach. This deep learning approach was trained with classical antibodies and V_H_Hs, as a large amount of data is needed to train the neural network and obtain relevant results. Its architecture is made by a convolutional neural network (CNN) with two 1D residual neural networks (ResNet). The first ResNet learns the frameworks and CDR hypervariable loops, while the second will apprehend inter-residue interactions. After a dropout layer to avoid overfitting, the last layer outputs C-alpha coordinates for a given input V_H_H sequence. Full backbone and side chains can then be constructed using Pulchra [169]. This program is available online (https://github.com/dina-lab3D/NanoNet, last accessed date: 13 February 2022). In our experience, it took less than 15 s to predict a full atomic structure of a V_H_H using a computer with 8 GB RAM, 4 CPUs and an Intel i5 processor. No particular library is required for the use of this program.

The assessment of NanoNet was done against AlphaFold 2 with 16 V_H_H deposited in the PDB in 2021, i.e., absent from AlphaFold 2 training. NanoNet performed better with a mean RMSD of 2.69 (±1.49) Å vs. 3.23 (±2.49) Å for AlphaFold 2 (and 1.57 (±0.41) Å vs. 2.04 (±2.09) Å on CDR3, reps.). Similar outcomes were obtained with 37 V_H_Hs when confronted with the Rosetta Antibody modelling suite. NanoNet performed better with a mean RMSD of 1.68 (±0.57) Å vs. 2.71 (±1.13) Å for Rosetta Antibody (and 2.99 (±1.48) Å vs. 5.73 (±2.33) Å on CDR3, resp.).

Hence, NanoNet appears to be a very interesting and promising new tool dedicated to V_H_H with excellent results. It has been, in particular, used to optimise CDR3 loop predictions (associated with the experimental data) in optimising anti-SARS-CoV-2 V_H_Hs [254].

## 3. Discussion

In the previous sections, we listed the different methodologies, provided their specificities and gave examples of V_H_H structural modelling. They are all very different and mainly used for docking purposes. However, it is highly difficult to precisely know the quality of each of these approaches/results. Indeed, they are all well-established approaches, but no real benchmark was performed until now, and the evaluations are limited to specific and extremely rare cases. A few examples are detailed hereafter.

(i) The dedicated development of V_H_H scripts for the RosettaAntibody modelling suite is as expected associated with a specific evaluation [211] but mainly focuses on classical RosettaAntibody modelling than another tool [207].
(ii) More recently, as Modeller [84] remains the most used comparative modelling approach, we evaluated the Modeller quality in V_H_H modelling [74]. Using 100 different V_H_Hs, different template selection strategies for comparative modelling using Modeller have been extensively assessed. This study analysed the conformational changes in both the FRs and CDRs using an original strategy of conformational discretisation based on a structural alphabet [255,256]. It showed that, often, multi-template is the best method to obtain a correct V_H_H model and that the DOPE score is a relevant measure to select this model [107]. Nonetheless, it remains difficult to propose satisfactory models for some V_H_Hs. Even sometimes, to use the closest V_H_H in terms of the RMSD is not always the best choice to obtain a good model, underlying the possibility for future improvement.
(iii) Finally, NanoNet has demonstrated its superiority over RosettaAntibody and AlphaFold 2 but with a limited number of examples (see the previous subsection) [253].

To better understand this complexity, we decided to evaluate and show a rather classical example of V_H_H modelling. We selected a structure of V_H_H that binds with lanthanides (PDB ID: 6XYF) [257] deposited in the PDB recent enough to never have been used in any of the previously cited approaches. 6XYF was obtained by X-ray crystallography with a very good resolution of 1.11 Å. Please note that this case is a “classical” case of structural prediction, i.e., representative of V_H_H modelling, as others were also tested. Eight different methods were carried out with six different software and online tools: Modeller with single-template and multi-template [84], ModWeb [258,259], SwissModel with the best sequence identity template and a second time with the best GMQE score [134], AlphaFold 2 [91], RoseTTAfold [251] and NanoNet [253].

At first, Modeller was used with the simplest approach, i.e., a single template. A template was selected with the highest sequence identity, i.e., a V_H_H domain (PDB ID: 5LMW chain A) used as a crystallisation chaperone for different constructs associated with the type IX secretion system from *Porphyromonas gingivalis* [260]. PDB 5LMW had a sequence identity of 89.9% with the target sequence. In addition, Modeller was used in a multiple-template approach with three templates. We decided to use this method because of its remarkable efficiency, as demonstrated in Reference [74]. Two new V_H_H structures were selected: anti-HIV V_H_H (PDB ID: 3R0M chain B) [261] and anti-HIV-1 gp120 V_H_H (PDB ID: 2XA3 chain A) [262], in addition to the 5LMW template. They also shared high sequence identity with 6XYF V_H_H: 89.3% and 88.9%, respectively. Each time, 100 models were generated, and the best model was selected using the DOPE score [107].

The ModWeb server was also used to model the 6XYF sequence. This online job provided one unique model based on a single template (PDB ID: 6XYM [257]) that had an extremely high sequence identity percentage (98%) with the target sequence. Indeed, this V_H_H came from the same lab as our test V_H_H [257].

These approaches can be easily compared to SwissModel. The first step with this method is to look at the best sequence identity. A V_H_H anti-SARS-CoV-1 (PDB ID: 6WAQ chain A) [54] was identified that shares 82.3% sequence identity with our target. An alternative model with the best GMQE score was built with a V_H_H anti-Vsig4 (PDB ID: 5IML chain B) with a sequence identity of 80.0%.

Regarding more advanced techniques, deep learning-based methodologies (RoseTTAfold, AlphaFold 2 and NanoNet) were used to model 6XYF. RoseTTAfold predicted five models. Their mean estimated RMS errors (in Å) per position were 1.10 ± 0.87, 1.13 ± 0.82, 1.12 ± 0.8, 1.12 ± 0.79 and 1.13 ± 0.84, respectively (displayed according to their ranks). AlphaFold 2 gave five models. These models had mean confidences (representing the accuracy in terms of the RMS) that were the following: 91.45 ± 10.38, 90.71 ± 11.05, 90.44 ± 11.54, 90.42 ± 11.83 and 89.99 ± 12.16. NanoNet produced only a single model for each query sequence.

**Figure 3:**
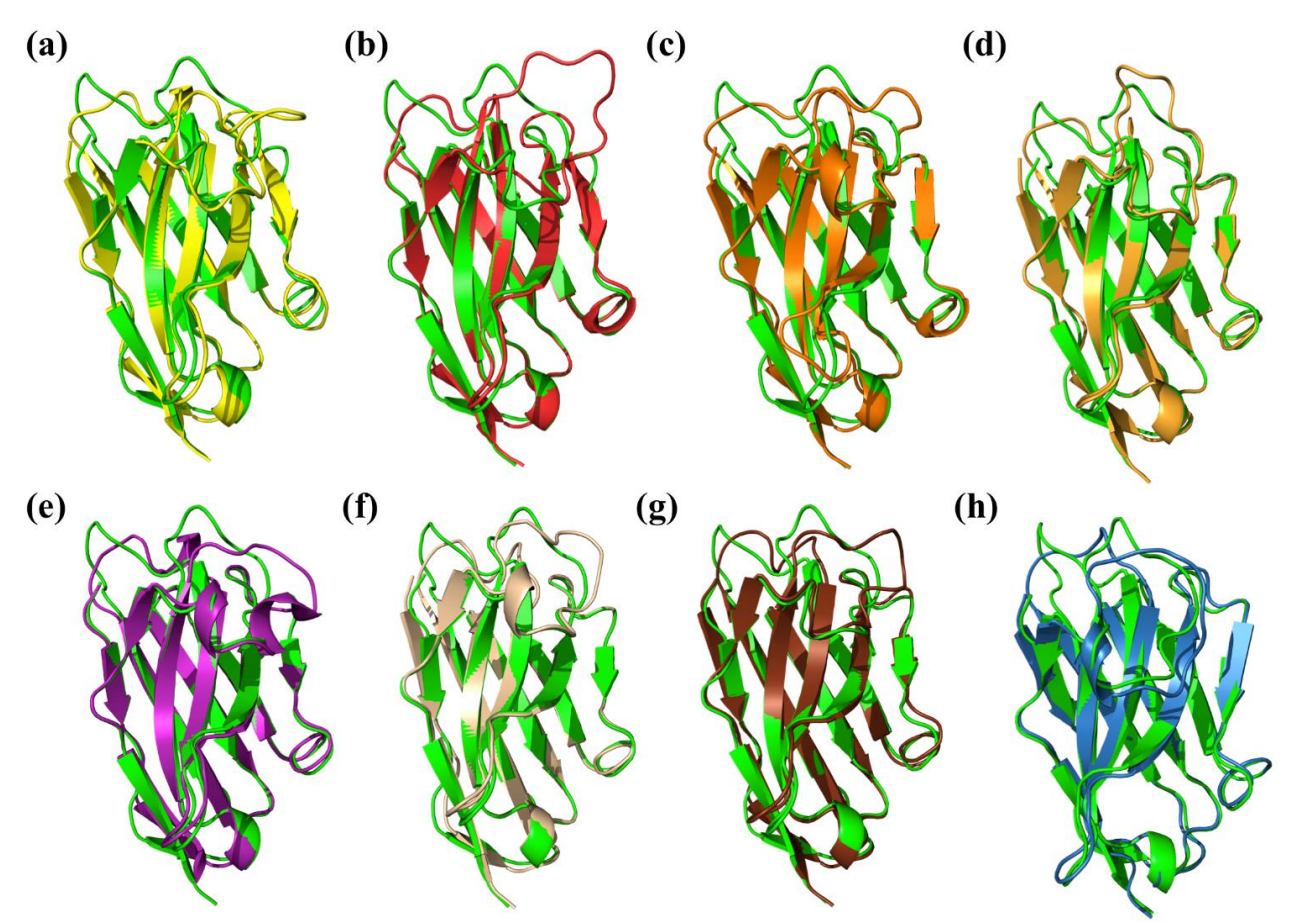
Superimposition of the V_H_H structural models with the X-ray structure of PDB ID: 6XYF. The V_H_H structure (in green) is superimposed with a structural model from the (**a**) Modeller best model for single-template (in yellow, RMSD = 2.28 Å, RMSD CDR3 = 3.80 Å), (**b**) Modeller best model for multi-template (in red, RMSD = 2.18 Å, RMSD CDR3 = 3.32 Å), (**c**) ModWeb (in orange, RMSD = 1.92 Å, RMSD CDR3 = 2.05 Å), (**d**) SwissModel best model with the best sequence identity (in yellow ochre, RMSD = 1.85 Å, RMSD CDR3 = 1.98 Å), (**e**) SwissModel best model with a GMQE score (in purple, RMSD = 2.05 Å, RMSD CDR3 = 2.75 Å), (**f**) AlphaFold 2 (in wheat, RMSD = 1.62 Å, RMSD CDR3 = 1.98 Å), (**g**) RoseTTAfold (in chocolate brown, RMSD = 1.87 Å, RMSD CDR3 = 2.56 Å) and (**h**) NanoNet (in blue, RMSD = 1.55 Å, RMSD CDR3 = 1.81 Å). Visualisation was performed using PyMOL [1,2,3].

Figure 3 shows every best structural model superimposed with the corresponding X-ray structure. The model with the best DOPE score obtained with a single-template Modeller approach showed a RMSD of 2.28 Å (see Figure 3a). This value was slightly improved with the addition of two close V_H_Hs, dropping to 2.18 Å (see Figure 3b). The template used by the ModWeb server resulted in a model with a RMSD value of 1.97 Å (Figure 3c), but we chose to discard this option, as this template was made by the same research group as the query [257]. Please note that, in our experience, ModWeb sometimes provides incomplete models. Actually, it depends on the pertinence of the identified template and the structural coverage. Concerning SwissModel, the best sequence identity model had the best RMSD of all the comparative approaches, with a value of 1.85 Å (see Figure 3d; the GMQE score and QMEANDisCo Global score [263] were 0.78 and 0.77 ± 0.07, respectively). For the SwissModel solution, considering the best GMQE, its RMSD was 2.05 Å (see Figure 3e; GMQE score = 0.81 and QMEANDisCo Global score = 0.79 ± 0.07).

The results for the deep learning-based techniques were also very relevant. The RoseTTAfold model ranked first had a RMSD of 1.87 Å (see Figure 3g) but was surpassed by the models from NanoNet and AlphaFold 2 with RMSDs of 1.55 Å (see Figure 3h) and 1.62 Å (see Figure 3f), respectively.

According to our case study based on 6XYF, it was demonstrated that NanoNet and AlphaFold 2 predicted the closest models to the experimental structure. This tendency was also found when CDR3 was specifically analysed. NanoNet and AlphaFold 2 ranked first and second (1.81 and 1.98 Å). SwissModel (with the best sequence identity) also performed well here, with a similar accuracy to AlphaFold 2, while the other methods were less efficient, with RMSDs ranging between 2.05 and 3.80 Å.

## 4. Conclusions and Perspectives

V_H_Hs have been known for almost 30 years, but in recent years, they have received a phenomenal revival of interest from an experimental point of view. Regarding bioinformatics, it has taken much longer to gain insight into their structures and functions due to inherent limitations. For example, the number of precise analyses of the sequence–structure relationship analyses of V_H_H domains, which is required to develop their in silico design and optimisation, is still limited [72,264,265].

There have also been some recent developments of web repositories dedicated to V_H_Hs. For instance, the Institute Collection and Analysis of Nanobodies (iCAN) has the first collected large number of V_H_H sequences coming from scientific publications and patents (http://ican.ils.seu.edu.cn, not operative at the moment of reviewing this paper, last try: 19 February 2022) [266]. Llamanade is a dedicated open-source computational pipeline for robust V_H_H humanisation [267]. More recently, an integrated nanobody database for immunoinformatics (INDI) lists V_H_Hs from all the major public outlets of biological sequences and patents (http://research.naturalantibody.com/nanobodies, last accessed date: 11 February 2022) from GenBank, next-generation sequencing repositories and structure databases and publications [268]. Even the famous SAbDab has an extension for V_H_H with SAbDab-nano (http://opig.stats.ox.ac.uk/webapps/newsabdab/nano/, last accessed date: 12 February 2022) [159]. All V_H_H structures could also be mined from the PDB.

**Figure 4:**
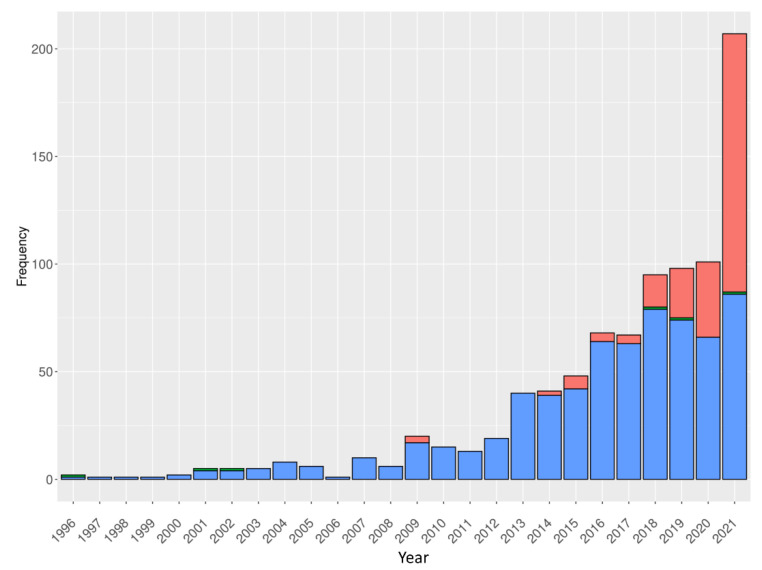
Number of V_H_H structures deposited in the PDB per year. Structures solved using different experimental methods are colour-coded accordingly (electron microscopy, solution NMR and X-ray diffraction has been represented in red, green and blue respectively). Data extracted from Reference [159].

**Figure A1:**
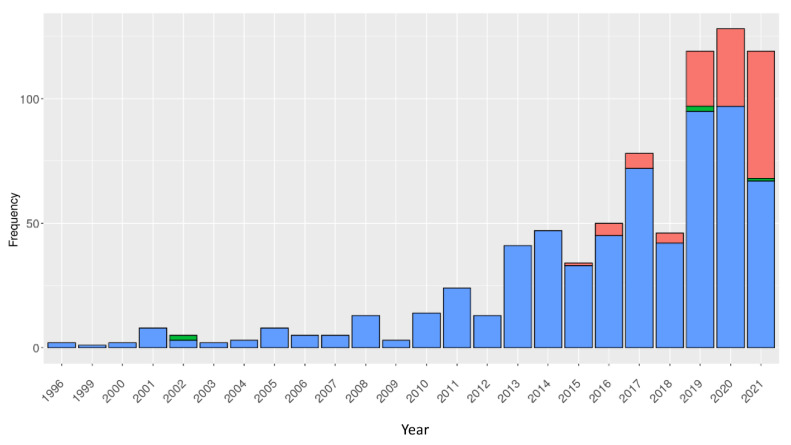
Number of V_H_H structures deposited in the PDB per year. Structures solved using different experimental methods are colour-coded accordingly (our own analysis) (electron microscopy, solution NMR and X-ray diffraction has been represented in red, green and blue respectively). It differs from Figure 4, as only nonmodified camelid V_H_Hs were analysed.

Concerning the structural features of V_H_Hs, specific developments are quite rare. We have shed light on the first optimised V_H_H structural modelling approach, NanoNet [253]. Since V_H_H docking is a key point in the development of those methods, a dedicated reranking approach of the V_H_H docking poses has been proposed with interest, namely NbX [269], but unfortunately, no tool is currently available to test it. Similarly, they can be positively integrated into epitope prediction approaches, as developed in Reference [270]. For a few years, the number of V_H_H structures deposited in the PDB greatly increased (see Figure 4 and Figure A1), which confirms the increasing interest in these small protein domains.

Besides 3D model production, there is also an interesting number of molecular dynamics simulations of V_H_Hs that are often used for their design optimisation [271,272,273,274,275]. Yet, there is no dedicated approach to globally apprehend their dynamics. An important point is that their CDRs are supposed to be variable and flexible. Moreover, molecular dynamics simulations are also interesting for going further with antibodies [276], as stated in his pioneer works by Nobel Prize Martin Karplus [277,278].

In this review, we have seen the limitations of our knowledge, i.e., the difficulty to apprehend the ability of each methodology for V_H_H modelling. The number of scenarios used to build V_H_H structural models is impressive and is often successful for apprehending atomistic-level epitopes–paratopes docking and experimental data. A more extensive modelling assessment would be good, especially with recent deep learning approaches such as AlphaFold 2 [91] and NanoNet [253], since multiple examples have shown how pertinent they might be.

## Data Availability

Not applicable.
